# Social Group Optimization–Assisted Kapur’s Entropy and Morphological Segmentation for Automated Detection of COVID-19 Infection from Computed Tomography Images

**DOI:** 10.1007/s12559-020-09751-3

**Published:** 2020-08-15

**Authors:** Nilanjan Dey, V. Rajinikanth, Simon James Fong, M. Shamim Kaiser, Mufti Mahmud

**Affiliations:** 1 Department of Information Technology, Techno India College of Technology, Kolkata, 700156 West Bengal India; 2 Department of Electronics and Instrumentation Engineering, St. Joseph’s College of Engineering, Chennai, 600119 India; 3 Department of Computer and Information Science, University of Macau, Taipa, China; 4 DACC Laboratory, Zhuhai Institutes of Advanced Technology of the Chinese Academy of Sciences, Zhuhai, China; 5 Institute of Information Technology, Jahangirnagar University, Savar, 1342 Dhaka Bangladesh; 6 Department of Computing & Technology, Nottingham Trent University, Clifton Lane, Nottingham NG11 8NS UK

**Keywords:** COVID-19 infection, CT scan image, Fused feature vector, KNN classifier, Segmentation and detection accuracy

## Abstract

The coronavirus disease (COVID-19) caused by a novel coronavirus, SARS-CoV-2, has been declared a global pandemic. Due to its infection rate and severity, it has emerged as one of the major global threats of the current generation. To support the current combat against the disease, this research aims to propose a machine learning–based pipeline to detect COVID-19 infection using lung computed tomography scan images (CTI). This implemented pipeline consists of a number of sub-procedures ranging from segmenting the COVID-19 infection to classifying the segmented regions. The initial part of the pipeline implements the segmentation of the COVID-19–affected CTI using social group optimization–based Kapur’s entropy thresholding, followed by k-means clustering and morphology-based segmentation. The next part of the pipeline implements feature extraction, selection, and fusion to classify the infection. Principle component analysis–based serial fusion technique is used in fusing the features and the fused feature vector is then employed to train, test, and validate four different classifiers namely Random Forest, K-Nearest Neighbors (KNN), Support Vector Machine with Radial Basis Function, and Decision Tree. Experimental results using benchmark datasets show a high accuracy (> 91%) for the morphology-based segmentation task; for the classification task, the KNN offers the highest accuracy among the compared classifiers (> 87%). However, this should be noted that this method still awaits clinical validation, and therefore should not be used to clinically diagnose ongoing COVID-19 infection.

## Introduction

Lung infection caused by coronavirus disease (COVID-19) has emerged as one of the major diseases and has affected over 8.2 million of the population globally^1^, irrespective of their race, gender, and age. The infection and the morbidity rates caused by this novel coronavirus are increasing rapidly [1, 2]. Due to its severity and progression rate, the recent report of the World Health Organization (WHO) declared it as pandemic [3]. Even though an extensive number of precautionary schemes have been implemented, the occurrence rate of COVID-19 infection is rising rapidly due to various circumstances.

The origin of COVID-19 is due to a virus called severe acute respiratory syndrome-coronavirus-2 (SARS-CoV-2) and this syndrome initially started in Wuhan, China, in December 2019 [4]. The outbreak of COVID-19 has appeared as a worldwide problem and a considerable amount of research works are already in progress to determine solutions to manage the disease infection rate and spread. Furthermore, the recently proposed research works on (i) COVID-19 infection detection [5–8], (ii) handling of the infection [9, 10], and (iii) COVID-19 progression and prediction [11–13] have helped get more information regarding the disease.

The former research and the medical findings discovered that COVID-19 initiates disease in the human respiratory tract and builds severe acute pneumonia. The existing research also confirmed that the premature indications of COVID-19 are subclinical and it necessitates a committed medical practice to notice and authenticate the illness. The frequent medical-grade analysis engages in a collection of samples from infected persons and sample supported examination and confirmation of COVID-19 using reverse transcription-polymerase chain reaction (RT-PCR) test and image-guided assessment employing lung computed tomography scan images (CTI), and the chest X-ray [14–17]. When the patient is admitted with COVID-19 infection, the doctor will initiate the treatment process to cure the patient using the prearranged treatment practice which will decrease the impact of pneumonia.

Usually, experts recommend a chain of investigative tests to identify the cause, position, and harshness of pneumonia. The preliminary examinations, such as blood tests and pleural-fluid assessment, are performed clinically to detect the severity of the infection [18–20]. The image-assisted methods are also frequently implemented to sketch the disease in the lung, which can be additionally examined by an expert physician or a computerized arrangement to recognize the severity of the pneumonia. Compared with chest X-ray, CTI is frequently considered due to its advantage and the 3-D view. The research work published on COVID-19 also confirmed the benefit of CT in detecting the disease in the respiratory tract and pneumonia [21–23].

Recently, more COVID-19 detection methods have been proposed for the progression stage identification of COVID-19 using the RT-PCR and imaging methods. Most of these existing works combined RT-PCR with the imaging procedure to confirm and treat the disease. The recent work of Rajinikanth et al. [8] developed a computer-supported method to assess the COVID-19 lesion using lung CTI. This work implemented few operator-assisted steps to achieve superior outcomes during the COVID-19 evaluation.

ML approaches are well-known for their capabilities in recognizing patterns in data. In recent years, ML has been applied to a variety of tasks including biological data mining [24, 25], medical image analysis [26], financial forecasting [27], trust management [28], anomaly detection [29, 30], disease detection [31, 32], natural language processing [33], and strategic game playing [34].

The presented work aims to:

- Propose a ML-driven pipeline to extract and detect the COVID-19 infection from lung CTI with an improved accuracy.
- Develop a procedural sequence for an automated extraction of the COVID-19 infection from a benchmark lung CTI dataset.
- Put forward an appropriate sequence of techniques, tri-level thresholding using social group optimization (SGO)-based Kapur’s entropy (KE) or SGO-KE, K-Means Clustering (KMC)-based separation, morphology-based segmentation to accurately extract COVID-19 infection from lung CTI.

A comparison of the extracted COVID-19 infection information from the CTI using the proposed pipeline with the ground truth (GT) images confirms the segmentation accuracy of the proposed method. The proposed pipeline achieves mean segmentation and classification accuracy of more than 91% and 87% respectively using 78 images from a benchmark dataset.

This research is arranged as follows; Section “Motivation” presents the motivation, Section “Methodology” represents the methodological details of the proposed scheme. Section “Results and Discussion” outlines the attained results and discussions. Section “Conclusion” depicts the conclusion of the present research work.

## Motivation

The proposed research work is motivated by the former image examination works existing in literature [35–38]. During the mass disease screening operation, the existing medical data amount will gradually increase and reduce the data burden; it is essential to employ an image segregation system to categorize the existing medical data into two or multi-class, and to assign the priority during the treatment implementation. The recent works in the literature confirm that the feature-fusion–based methods will improve the classification accuracy without employing the complex methodologies [39–41]. Classification task implemented using the features of the original image and the region-of-interest (ROI) offered superior result on some image classification problems and this procedure is recommended when the similarity between the normal and the disease class images is more [24, 26, 31, 42, 43]. Hence, for the identical images, it is necessary to employ a segmentation technique to extract the ROI from the disease class image with better accuracy [26]. Finally, the fused features of the actual image and the ROI are fused to attain enhanced classification accuracy.

## Methodology

This section of the work presents the methodological details of the proposed scheme. Like the former approaches, this work also implemented two different phases to improve the detection accuracy.

### Proposed Pipeline

**Fig. 1:**
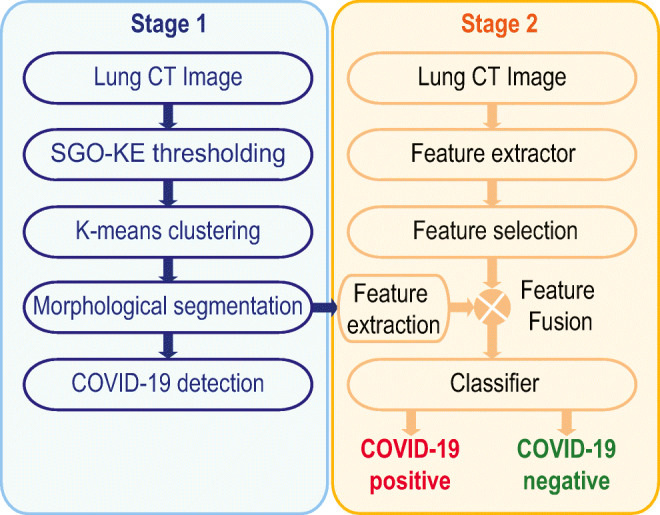
The number of image processing stages implemented in the proposed work

This work consists of the following two stages as depicted in Fig. 1. These are:

- Implementation of an image segmentation method to extract the COVID-19 infection,
- Execution of a ML scheme to classify the considered lung CTI database into normal/COVID-19 class.

The details of these two stages are given below:

#### Stage 1:

**Fig. 2:**
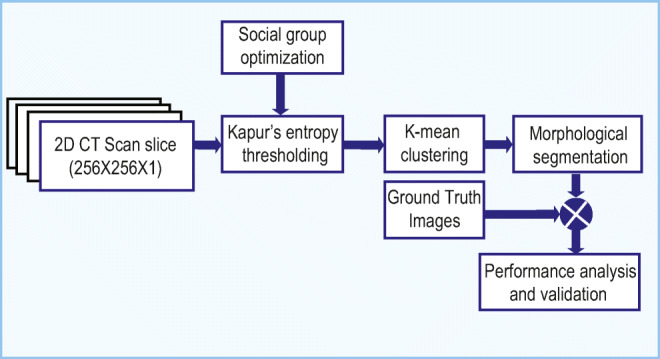
Image segmentation framework to extract COVID-19 infection from 2D lung CT scan image

Figure 2 depicts the image processing system proposed to extract the pneumonia infection in the lung due to COVID-19. Initially, the required 2D slices of the lung CTI are collected from an open-source database [44]. All the collected images are resized into 256 × 256 × 1 pixels and the normalized images are then considered for evaluation. In this work, SGO-KE–based tri-level threshold is initially applied to enhance the lung section (see “Social Group Optimization and Kapur’s Function” for details). Then, KMC is employed to segregate the thresholded image into background, artifact, and the lung segment. The unwanted lung sections are then removed using a morphological segmentation procedure and the extracted binary image of the lung is then compared with its related GT provided in the database. Finally, the essential performance measures are computed and based on which the performance of the proposed COVID-19 system is validated.

#### Stage 2:

**Fig. 3:**
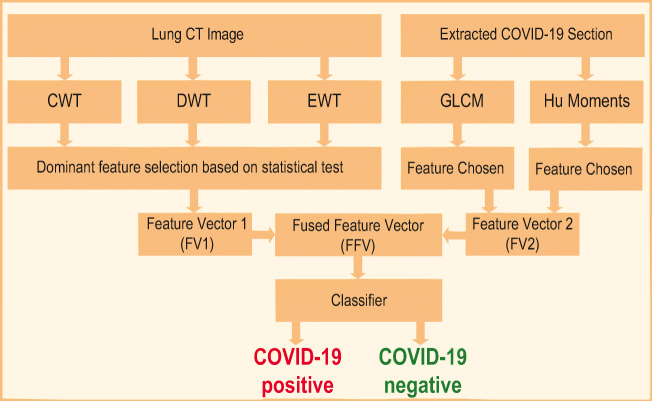
Proposed ML scheme to detect COVID-19 infection

Figure 3 presents the proposed ML scheme to separate the considered lung CTI into normal/COVID-19 class. This system is constructed using two different images, such as (i) the original test image (normal/COVID-19 class) and (ii) the binary form of the COVID-19 section. The various procedures existing in the proposed ML scheme are depicted in Fig. 3.

#### Segmentation of COVID-19 Infection

This procedure is implemented only for the CTI associated with the COVID-19 pneumonia infection. The complete details on various stages involved in this process are depicted in Fig. 1. The series of procedures implemented in this figure are used to extract the COVID-19 infection from the chosen test image with better accuracy. The pseudo-code of the implemented procedure is depicted in Algorithm 1.

**Figure:**
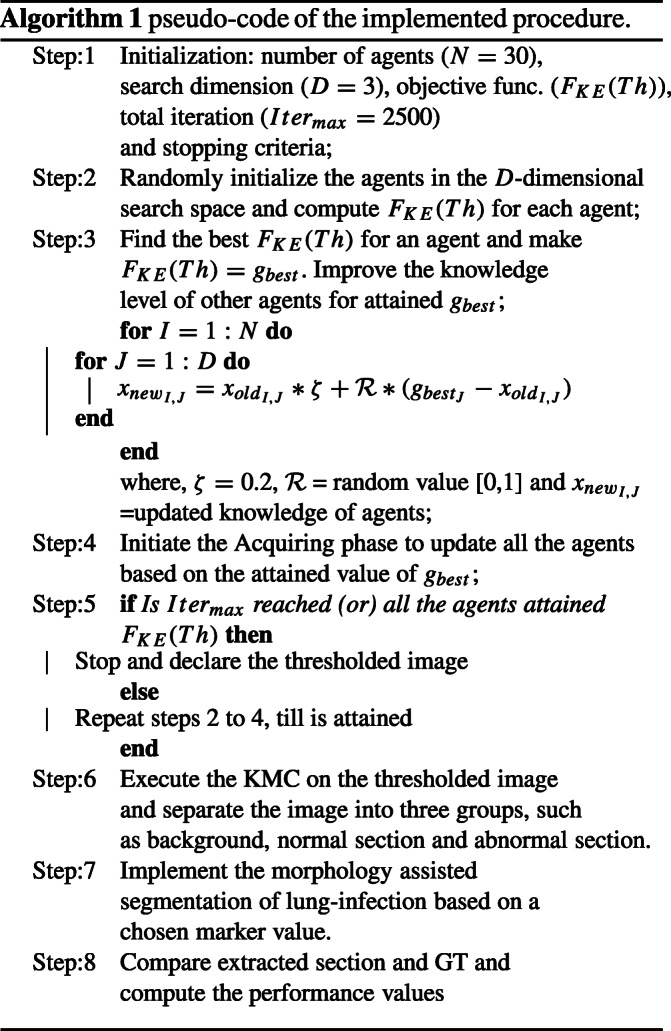

##### Image Thresholding

Initially, the enhancement of the infected pneumonia section is achieved by implementing a tri-level threshold based on SGO and the KE. In this operation, the role of the SGO is to randomly adjust the threshold value of the chosen image until KE is maximized. The threshold which offered the maximized KE is considered as the finest threshold. The related information on the SGO-KE implemented in this work can be found in [45]. The SGO parameters discussed in Dey et al. [46] are considered in the proposed work to threshold the considered CTI.

##### Social Group Optimization and Kapur’s Function

SGO is a heuristic technique proposed by Satapathy and Naik [47] by mimicking the knowledge sharing concepts in humans. This algorithm employs two phases, such as (i) enhancing phase to coordinate the arrangement of people (agents) in a group, and the (ii) knowledge gaining phase: which allows the agents to notice the finest solution based on the task. In this paper, an agent is considered a social population who is generated based on the features/parameters.

The mathematical description of the SGO is defined as: let *X*_*I*_ denote the original knowledge of agents of a group with dimension *I* = 1, 2, ... , *N*. If the number of variables to be optimized is represented as *D*, then the initial knowledge can be expressed as *X*_*I*_ = (*x*_*I*1_, *x*_*I*2_,... *x*_*ID*_). For a chosen problem, the objective function can be defined as *F*_*J*_, with *J* = 1, 2, ... , *N*.

The updated function in SGO is;

1 $$ X_{new_{I,J}}=X_{old_{I,J}} \zeta + R (g_{best_{J}}-X_{old_{I,J}} ) $$

where $X_{new_{i,j}}$ is the original knowledge, $X_{old_{i,j}}$ is the updated knowledge, *ζ* denotes self-introspection parameter (assigned as 0.2), *R* is the random number [0,1], and $g_{best_{j}}$ is the global best knowledge.

In this work, the SGO is employed to find the optimal threshold by maximizing the KE value and this operation is defined below:

Entropy in an image is the measure of its irregularity and for a considered image, Kapur’s thresholding can be used to identify the optimal threshold by maximizing its entropy value.

Let *Th* = [*t*_1_, *t*_2_, ... , *t*_*n*− 1_] denote the threshold vector of the chosen image of a fixed dimension and assume this image has *L* gray levels (0 to *L* − 1) with a total pixel value of *Z*. If*f*() represents the frequency of *j*-th intensity level, then the pixel distribution of the image will be:

2 $$ Z=f(0)+f(1)+...+f(L-1). $$

If the probability of *j*-th intensity level is given by:

3 $$ P_{j}=f(j)/Z. $$

Then, during the threshold selection, the pixels of image are separated into *Th* + 1 groups according to the assigned threshold value. After disconnection of the images as per the selected threshold, the entropy of each cluster is separately computed and combined to get the final entropy as follows:

The KE to be maximized is given by Eq. 14:

4 $$ KE_{max}=F_{KE}(Th)=\sum\limits_{i=1}^{n}{G_{i}^{C}}. $$

For a tri-level thresholding problem, the expression will be given by Eq. 5:

5 $$ f(t_{1},t_{2},t_{3})=\sum\limits_{i=1}^{3}{G_{i}^{C}}. $$

where *G*_*i*_ is the entropy given by:

6 $$ \begin{array}{@{}rcl@{}} {G_{1}^{C}}&=&\sum\limits_{j=1}^{t_{1}}\frac{{P_{j}^{C}}}{{w_{0}^{C}}}\ln\left( \frac{{P_{j}^{C}}}{{w_{0}^{C}}}\right), \end{array} $$

7 $$ \begin{array}{@{}rcl@{}} {G_{2}^{C}}&=&\sum\limits_{j=t_{1}}^{t_{2}}\frac{{P_{j}^{C}}}{{w_{1}^{C}}}\ln\left( \frac{{P_{j}^{C}}}{{w_{1}^{C}}}\right), \end{array} $$

8 $$ \begin{array}{@{}rcl@{}} {G_{3}^{C}}&=&\sum\limits_{j=t_{2}}^{t_{3}}\frac{{P_{j}^{C}}}{{w_{2}^{C}}}\ln\left( \frac{{P_{j}^{C}}}{{w_{2}^{C}}}\right), \end{array} $$

where,

${P_{j}^{C}}$ is the probability distribution for intensity, *C* is the image class (*C* = 1 for the grayscale image), and $w_{i-1}^{C}$ is the probability occurrence.

During the tri-level thresholding, a chosen approach is employed to find the *F*_*KE*_(*Th*) by randomly varying the thresholds (*Th* = {*t*_1_, *t*_2_, *t*_3_} ). In this research, the SGO is employed to adjust the thresholds to find the *F*_*KE*_(*Th*).

##### Segmentation Based on KMC and Morphological Process

The COVID-19 infection from the enhanced CTI is then separated using the KMC technique and this approach helps segregate the image into various regions [48]. In this work, the enhanced image is separated into three sections, such as the background, normal image section, and the COVID-infection. The essential information on KMC and the morphology-based segmentation can be found in [49]. The extracted COVID-19 is associated with the artifacts; hence, morphological enhancement and segmentation discussed in [49, 50] are implemented to extract the pneumonia infection, with better accuracy.

KMC helps split *u*-observations into K-groups. For a given set of observations with dimension “*d*,” KMC will try to split them into *K*-groups; *Q*(*Q*_1_, *Q*_2_, ... , *Q*_*K*_) for (*K* ≤ *u*) to shrink the within-cluster sum of squares as depicted by Eq. 9:

9 $$ \arg \min_{Q}\sum\limits_{i=1}^{K}||O_{i}-\mu_{i}||^{2}=\arg \min_{Q}\sum\limits_{i=1}^{K}|Q_{i}|Var(Q_{i}) $$

where *O* is the number of observations, *Q* is the number of splits, and *μ*_*j*_ is the mean of points in *Q*_*i*_.

##### Performance Computation

The outcome of the morphological segmentation is in the form of binary and this binary image is then compared against the binary form of the GT and then the essential performance measures, such as accuracy, precision, sensitivity, specificity, and F1-score, are computed. A similar procedure is implemented on all the 78 images existing in the benchmark COVID-19 database and the mean values of these measures are then considered to confirm the segmentation accuracy of the proposed technique. The essential information on these measures is clearly presented in [51, 52].

#### Implementation of Machine Learning Scheme

The ML procedure implemented in this research is briefed in this section. This scheme implements a series of procedures on the original CTI (normal/COVID-19 class) and the segmented binary form of the COVID-19 infection as depicted in Fig. 2. The main objective of this ML scheme is to segregate the considered CTI database into normal/COVID-19 class images. The process is shown in algorithm 2. 
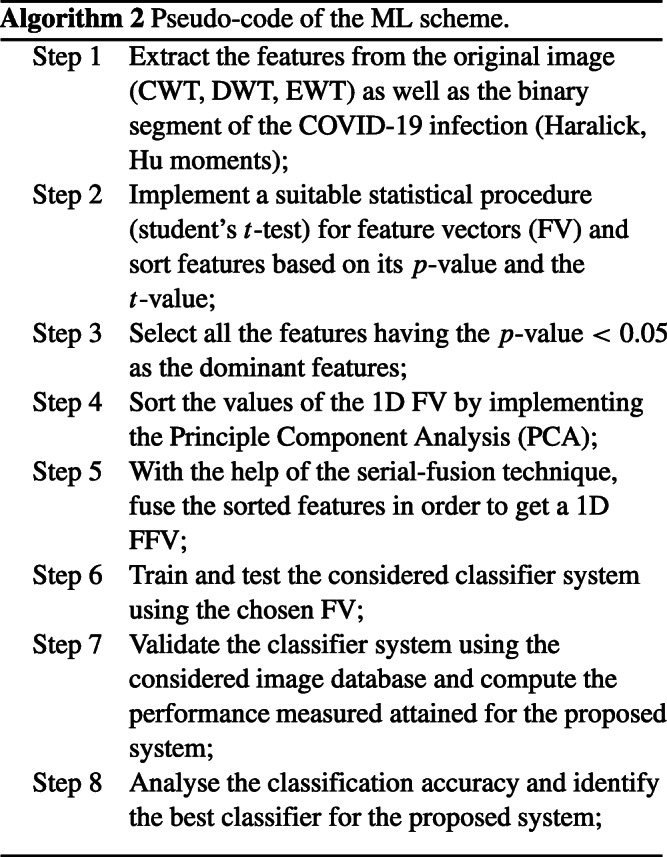


##### Initial Processing

This initial processing of the considered image dataset is individually executed for the test image and the segmented COVID-19 infection. The initial processing involves extracting the image features using a chosen methodology and formation of a one-dimensional FV using the chosen dominant features.

##### Feature Vector 1 (FV1):

The accuracy of disease detection using the ML technique depends mainly on the considered image information. In the literature, a number of image feature extraction procedures are discussed to examine a class of medical images [35–37, 39–42]. In this work, the well-known image feature extraction methods, such as Complex-Wavelet-Transform (CWT) and Discrete-Wavelet-Transform (DWT) as well as Empirical-Wavelet-Transform (EWT) are considered in 2-D domain to extract the features of the normal/COVID-19 class grayscale images. The information on the CWT, DWT, and EWT are clearly discussed in the earlier works [52]. After extracting the essential features using these methods, a statistical evaluation and Student’s *t* test–based validation is implemented to select the dominant features to create the essential FVs, such as *FV*_*CWT*_ (34 features), *FV*_*DWT*_ (32 features), and *FV*_*EWT*_ (3 features) which are considered to get the principle FV1 set (FV1 = 69 features) by sorting and arranging these features based on its *p* value and *t* value. The feature selection process and FV1 creation are implemented as discussed in [52].

- CWT: This function was derived from the Fourier transform and is represented using complex-valued scaling function and complex-valued wavelet as defined below;
  10 $$ \psi_{C}(t)=\psi_{R}(t)+\psi_{I}(t) $$
  where *ψ*_*C*_(*t*), *ψ*_*R*_(*t*), and *ψ*_*I*_(*t*) represent the complex, real, and image parts respectively.
- DWT: This approach evaluates the non-stationary information. When a wavelet has the function *ψ*(*t*) ∈ *W*^2^(*r*), then its DWT (denoted by *DWT*(*a*, *b*)) can be written as:
  11 $$ DWT(a,b)=\frac{1}{\sqrt{2^{a}}} {\int}_{-\infty}^{\infty}x(t)\psi^{*}\left( \frac{t-b2^{a}}{2^{a}}\right) dt $$
  where *ψ*(*t*) is the principle wavelet, the symbol ∗ denotes the complex conjugate, *a* and *b* (*a*, *b* ∈ *R*) are scaling parameters of dilation and transition respectively.
- EWT: The Fourier spectrum of EWT of range 0 to *π* is segmented into *M* regions. Each limit is denoted as *ω*_*m*_ (where *m* = 1, 2, ... , *M*) in which the starting limit is *ω*_0_ = 0 and final limit is *ω*_*M*_ = *π*. The translation phase *T*_*m*_ centered around *ω*_*m*_ has a width of 2Φ_*m*_ where Φ_*m*_ = *λω*_*m*_ for 0 < *λ* < 1. Other information on EWT can be found in [53].

##### Feature Vector 2 (FV2):

The essential information from the binary form of COVID-19 infection image is extracted using the feature extraction procedure discussed in Bhandary et al. [35] and this work helped get the essential binary features using the Haralick and Hu technique. This method helps get 27 numbers of features (*F*_*Haralick*_ = 18 features and *F*_*Hu*_ = 9 features) and the combination of these features helped get the 1D FV2 (FV2 = 27 features).

- *Haralick features*: Haralick features are computed using a Gray Level Co-occurrence Matrix (GLCM). GLCM is a matrix, in which the total rows and columns depend on the gray levels (*G*) of the image. In this, the matrix component *P*(*i*, *j*|Δ*x*,Δ*y*) is the virtual frequency alienated by a pixel space (Δ*x*,Δ*y*). If *μ*_*x*_ and *μ*_*y*_ represent the mean and *σ*_*x*_ and *σ*_*y*_ represent the standard deviation of *P*_*x*_ and *P*_*y*_, then:
  12 $$ \begin{array}{@{}rcl@{}} \mu_{x}&=&{\sum}_{i=0}^{G-1}iP_{x}(i),\\ \mu_{y}&=&{\sum}_{j=0}^{G-1}jP_{y}(j),\\ \sigma_{x}&=&{\sum}_{i=0}^{G-1}(P_{x}(i)-\mu_{x}(i))\\ \sigma_{y}&=&{\sum}_{j=0}^{G-1}(P_{y}(j)-\mu_{y}(j)). \end{array} $$
  where *P*_*x*_(*i*) and *P*_*y*_(*j*) matrix components during the *i*-th and *j*-th entries, respectively.
  These parameters can be used to extract the essential texture and shape features from the considered grayscale image.
- *Hu moments*: For a two-dimensional (2D) image, the 2D (*i* + *j*)-th order moments can be defined as;
  13 $$ M_{ij}={\int}_{-\infty}^{\infty}{\int}_{-\infty}^{\infty}x^{i}y^{j}f(x,y)dxdy $$
  for *i*, *j* = 0, 1, 2,... If the image function *f*(*x*, *y*) is a piecewise continuous value, then the moments of all order exist and the moment sequence *M*_*ij*_ is uniquely determined. Other information on Hu moments can be found in [35].

##### Fused Feature Vector (FFV:)

In this work, the original test image helped get the FV1 and the binary form of the COVID-19 helps get the FV2. To implement a classifier, it is essential to have a single feature vector with a pre-defined dimension.

In this work, the FFV based on the principle component analysis (PCA) is implemented to attain a 1D FFV (69 + 27 = 96 features) by combining the FV1 and FV2, and this feature set is then considered to train, test, and validate the classifier system implemented in this study. The complete information on the feature fusion based on the serial fusion can be found in [35, 54].

##### Classification

Classification is one of the essential parts in a verity of ML and deep learning (DL) techniques implemented to examine a class of medical datasets. The role of the classifier is to segregate the considered medical database into two-class and multi-class information using the chosen classifier system. In the proposed work, the classifiers, such as Random-Forest (RF), Support Vector Machine-Radial Basis Function (SVM-RBF), K-Nearest Neighbors (KNN), and Decision Tree (DT), are considered. The essential information on the implemented classifier units can be found in [35, 36, 45, 52]. A fivefold cross-validation is implemented and the best result among the trial is chosen as the final classification result.

##### Validation

From the literature, it can be noted that the performance of the ML and DL-based data analysis is normally confirmed by computing the essential performance measures [35, 36]. In this work, the common performance measures, such as accuracy (4), precision (15), sensitivity (16), specificity (17), F1-score (18), and negative predictive value (NPV) (19) computed.

The mathematical expression for these values is as follows:

14 $$ \text{Accuracy}=\frac{(T_{P}+T_{N})}{(T_{P}+T_{N}+F_{P}+F_{N} )} $$

15 $$ \text{Precision}=\frac{T_{P}}{(T_{P}+F_{P} )} $$

16 $$ \text{Sensitivity}=\frac{T_{P}}{(T_{P}+F_{N})} $$

17 $$ \text{Specificity}=\frac{T_{N}}{(T_{N}+F_{P})} $$

18 $$ \text{F1-Score}=\frac{2T_{P}}{(2T_{P}+F_{N}+F_{P})} $$

19 $$ \text{NPV}=\frac{T_{N}}{(T_{N}+F_{N})} $$

where *T*_*P*_= true positive, *T*_*N*_= true negative, *F*_*P*_= false positive, and *F*_*N*_=false negative.

### COVID-19 Dataset

The clinical-level diagnosis of the COVID-19 pneumonia infection is normally assessed using the imaging procedure. In this research, the lung CTI are considered for the examination and these images are resized into 256 × 256 × 1 pixels to reduce the computation complexity. This work considered 400 grayscale lung CTI (200 normal and 200 COVID-19 class images) for the assessment. This research initially considered the benchmark COVID-19 database of [44] for the assessment. This dataset consists of 100 2D lung CTI along with its GT; and in this research, only 78 images are considered for the assessment and the remaining 22 images are discarded due to its poor resolution and the associated artifacts. The remaining COVID-19 CTI (122 images) are collected from the Radiopaedia database [55] from cases 3 [56], 8 [57], 23 [58], 10 [59], 27 [60] 52 [61], 55 [62], and 56 [63].

**Fig. 4:**
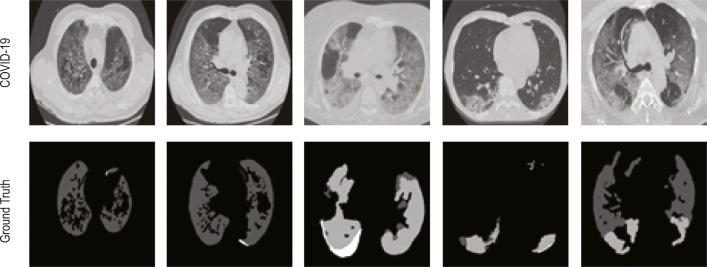
Sample test images of COVID-19 and the GT collected from [24]

**Fig. 5:**
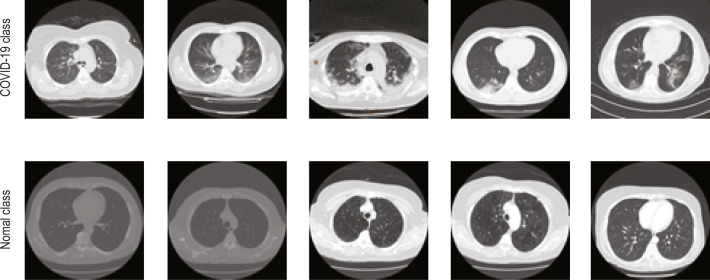
Sample test images of COVID-19 and normal group

The normal class images of the 2D lung CTI have been collected from The Lung Image Database Consortium-Image Database Resource Initiative (LIDC-IDRI) [64–66] and The Reference Image Database to Evaluate therapy Response-The Cancer Imaging Archive (RIDER-TCIA) [66, 67] database and the sample images of the collected dataset are depicted in Figs. 4 and 5. Figure 4 presents the test image and the related GT of the benchmark CTI. Figure 5 depicts the images of the COVID-19 [55] and normal lung [64, 67] CTI considered for the assessment.

## Results and Discussion

**Fig. 6:**
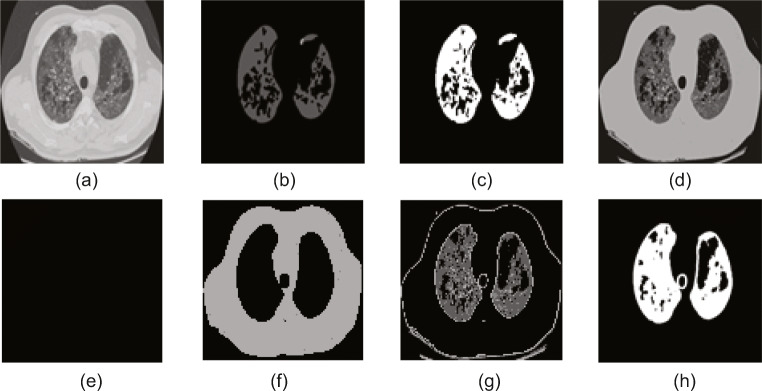
Results attained with the benchmark COVID-19 database. **a** Sample test image. **b** FT image. **c** Binary GT. **d** SGO-KE thresholded image. **e** Background. **f** Artifact. **g** Lung section. **h** Segmented COVID-19 infection

The experimental results obtained in the proposed work are presented and discussed in this section. This developed system is executed using a workstation with the configuration: Intel i5 2.GHz processor with 8GB RAM and 2GB VRAM equipped with the MATLAB (www.mathworks.com). Experimental results of this study confirm that this scheme requires a mean time of 173 ± 11 s to process the considered CTI dataset and the processing time can be improved by using a workstation with higher computational capability. The advantage of this scheme is it is a fully automated practice and will not require the operator assistance during the execution. The proposed research initially executes the COVID-19 infection segmentation task using the benchmark dataset of [44]. The results attained using a chosen trial image are depicted in Fig. 6. Figure 6a depicts the sample image of dimension 256 × 256 × 1 and Fig. 6b and c depict the actual and the binary forms of the GT image. The result attained with the SGO-KE-based tri-level threshold is depicted in Fig. 6d. Later, the KMC is employed to segregate Fig. 6d into three different sections and the separated images are shown in Fig. 6e–g. Finally, a morphological segmentation technique is implemented to segment the COVID-19 infection from Fig. 6g and the attained result is presented in Fig. 6h. After extracting the COVID-19 infection from the test image, the performance of the proposed segmentation method is confirmed by implementing a comparative examination between the binary GT existing in Fig. 6c with Fig. 6h and the essential performance values are then computed based on the pixel information of the background (0) and the COVID-19 section (1). For this image, the values attained are *T*_*P*_ = 5865 pixels, *F*_*P*_ = 306, *T*_*N*_ = 52572, and *F*_*N*_ = 1949, and these values offered accuracy = 96.28%, precision = 95.04%, sensitivity = 75.06%, specificity = 99.42%, F1-score = 83.88%, and NPV = 96.43%.

**Fig. 7:**
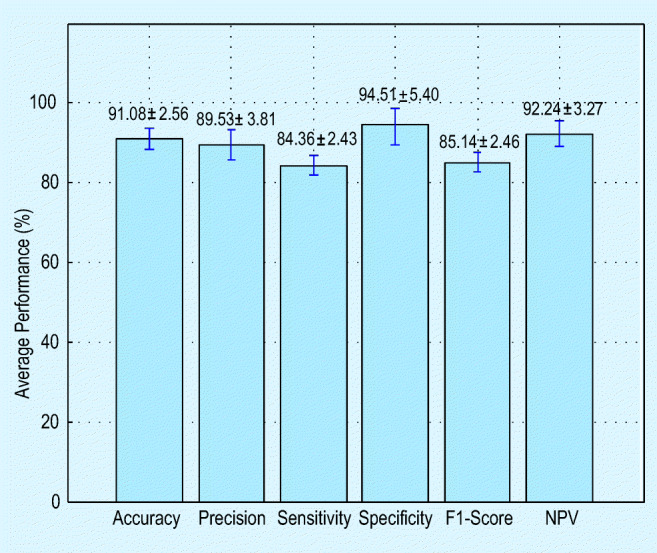
Mean performance measure attained with the proposed COVID-19 segmentation procedure

A similar procedure is implemented for other images of this dataset and means performance measure attained for the whole benchmark database (78 images) is depicted in Fig. 7. From this figure, it is evident that the segmentation accuracy attained for this dataset is higher than 91%, and in the future the performance of the proposed segmentation method can be validated against other thresholding and segmentation procedures existing in the medical imaging literature.

The methodology depicted in Fig. 3 is then implemented by considering the entire database of the CTI prepared in this research work. This dataset consists of 400 grayscale images with dimension 256 × 256 × 1 pixels and the normal/COVID-19 class images have a similar dimension to confirm the performance of the proposed technique. Initially, the proposed ML scheme is implemented by considering only the grayscale image features (FV1) with a dimension 1 × 69 and the performance of the considered classifier units, such as RF, KNN, SVM-RBF, and DT, is computed. During this procedure, 70% of the database (140 + 140 = 280 images) are considered for training and 30% (60 + 60 = 120 images) are considered for testing. After checking its function, each classifier is separately validated by using the entire database and the attained results are recorded. Here, a fivefold cross-validation is implemented for each classifier and the best result attained is considered as the final result. The obtained results are depicted in Table 1 (the first three rows). The results reveal that the classification accuracy attained with SVM-RBF is superior (85%) compared with the RF, KNN, and DT. Also, the RF technique helped get the better values of the sensitivity and NPV compared with other classifiers.

**Table 1:** Disease detection performance attained with the proposed ML scheme

Features	Classifier	TP	FN	TN	FP	Acc. (%)	Prec. (%)	Sens. (%)	Spec. (%)	F1-Sc. (%)	NPV (%)
FV1 (1×69)	RF	163	37	172	28	83.75	85.34	*81.50*	86.00	83.37	*82.30*
	KNN	159	41	177	23	84.00	87.36	79.50	88.50	83.24	81.19
	SVM-RBF	161	39	179	21	*85.00*	*88.46*	80.50	*89.50*	*84.29*	82.11
	DT	160	40	168	32	82.00	83.33	80.00	84.00	81.63	80.77
FFV (1×96)	RF	169	31	178	22	86.75	*88.48*	84.50	*89.00*	86.45	85.17
	KNN	178	22	173	27	*87.75*	86.83	*89.00*	86.50	*87.90*	*88.72*
	SVM-RBF	172	28	177	23	87.25	88.20	86.00	88.50	87.09	86.34
	DT	174	26	172	28	86.50	86.14	87.00	86.00	86.57	86.89

*TP*, true positive; *FN*, false negative; *TN*, true negative; *FP*, false positive; *Acc.*, accuracy; *Prec.*, precision; *Sens.*, sensitivity; *Spec.*, specificity; *F1-Sc.*, F1-score; *NPV*, negative predictive value, italicized values indicate the best performance.

**Fig. 8:**
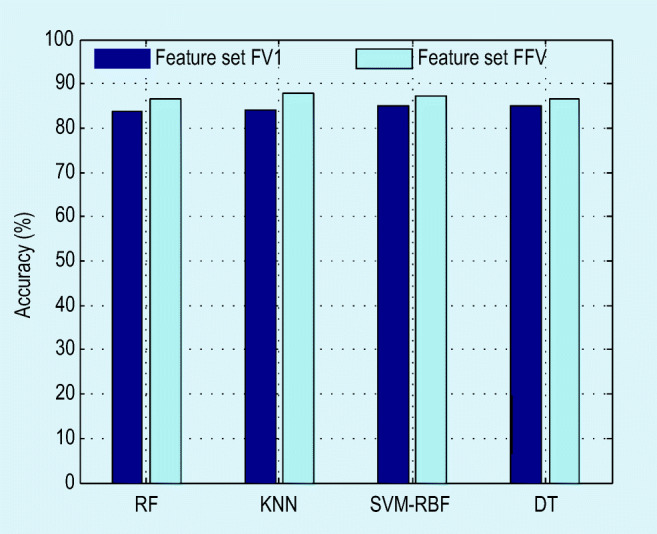
Detection accuracy attained in the proposed system with various classifiers

To improve the detection accuracy, the feature vector size is increased by considering the FFV (1 × 96 features) and a similar procedure is repeated. The obtained results (as in Table 1, bottom three rows) with the FFV confirm that the increment of features improves the detection accuracy considerably and the KNN classifier offers an improved accuracy (higher than 87%) compared with the RF, SVM-RBF, and DT. The precision and the F1-score offered by the RF are superior compared with the alternatives. The experimental results attained with the proposed ML scheme revealed that this methodology helps achieve better classification accuracy on the considered lung CTI dataset. The accuracy attained with the chosen classifiers for FV1 and FFV is depicted in Fig. 8. The future scope of the proposed method includes (i) implementing the proposed ML scheme to test the clinically obtained CTI of COVID-19 patients; (ii) enhancing the performance of implemented ML technique by considering the other feature extraction and classification procedures existing in the literature; and (iii) implementing and validating the performance of the proposed ML with other ML techniques existing in the literature; and (iv) implementing an appropriate DL architecture to attain better detection accuracy on the benchmark as well as the clinical grade COVID-19 infected lung CTI.

## Conclusion

The aim of this work has been to develop an automated detection pipeline to recognize the COVID-19 infection from lung CTI. This work proposes an ML-based system to achieve this task. The proposed system executed a sequence of procedures ranging from image pre-processing to the classification to develop a better COVID-19 detection tool. The initial part of the work implements an image segmentation procedure with SGO-KE thresholding, KMC-based separation, morphology-based COVID-19 infection extraction, and a relative study between the extracted COVID-19 sections with the GT. The segmentation assisted to achieve an overall accuracy higher than 91% on a benchmark CTI dataset. Later, an ML scheme with essential procedures such as feature extraction, feature selection, feature fusion, and classification is implemented on the considered data, and the proposed scheme with the KNN classifier achieved an accuracy higher than 87%.

## Footnotes

1 https://www.worldometers.info/coronavirus/, as of June 17, 2020
